# Decision making around living and deceased donor kidney transplantation: a qualitative study exploring the importance of expected relationship changes

**DOI:** 10.1186/1471-2369-13-103

**Published:** 2012-09-07

**Authors:** Ingrid B de Groot, Karen Schipper, Sandra van Dijk, Paul J M van der Boog, Anne M Stiggelbout, Andrzej G Baranski, Perla J Marang-van de Mheen

**Affiliations:** 1 Department of Medical Decision Making, Leiden University Medical Center, Leiden, The Netherlands; 2 Department of Medical Humanities, EMGO institute, VU Medical Center, Amsterdam, The Netherlands; 3 Department of Medical Psychology, Leiden University Medical Center, Leiden, The Netherlands; 4 Department of Nephrology, Leiden University Medical Center, Leiden, The Netherlands; 5 Department of Transplantation Surgery, Leiden University Medical Center, Leiden, The Netherlands

**Keywords:** Decision making, Donor-recipient relationship, Expectations, Kidney transplantation

## Abstract

**Background:**

Limited data exist on the impact of living kidney donation on the donor-recipient relationship. Purpose of this study was to explore motivations to donate or accept a (living donor) kidney, whether expected relationship changes influence decision making and whether relationship changes are actually experienced.

**Methods:**

We conducted 6 focus groups in 47 of 114 invited individuals (41%), asking retrospectively about motivations and decision making around transplantation. We used qualitative and quantitative methods to analyze the focus group transcripts.

**Results:**

Most deceased donor kidney recipients had a potential living donor available which they refused or did not want. They mostly waited for a deceased donor because of concern for the donor’s health (75%). They more often expected negative relationship changes than living donor kidney recipients (75% vs. 27%, p = 0.01) who also expected positive changes. Living donor kidney recipients mostly accepted the kidney to improve their own quality of life (47%). Donors mostly donated a kidney because transplantation would make the recipient less dependent (25%). After transplantation both positive and negative relationship changes are experienced.

**Conclusion:**

Expected relationship changes and concerns about the donor’s health lead some kidney patients to wait for a deceased donor, despite having a potential living donor available. Further research is needed to assess whether this concerns a selected group.

## Background

Due to a shortage of organ donors, kidney transplantation using living donors is increasingly performed in the Netherlands. Quality of life of most living donors is better than or equal to the general population [1,2] and returns to pre-donation levels after donation [3,4]. Receiving a living donor kidney has clear advantages in terms of improved graft survival [5], but disadvantages may be psychological, for example that it influences the donor-recipient relationship.

Research regarding the effect of living kidney donation on the donor-recipient relationship has mostly been performed from the donor’s perspective [6-8]. Although the relationship deteriorated in some cases [9], no changes or an improved relationship are usually reported by donors [6,7], without any further specification. Thus, it is unknown which aspects of the relationship deteriorated or improved.

The recipient’s perspective has received less attention. Recipients may possibly feel they owe the donor something, hesitate to accept a kidney the donor may need later in life, or are afraid to disappoint the donor if the kidney would not function properly [6,8]. The recipient is likely to perceive this having an impact on the relationship, without the donor knowing or recognising this effect (so that it is not reported in studies focussing on the donor’s perspective).

Similar cognitions and fears about possible relationship changes may influence the recipient’s decision making when accepting a kidney offer. For example, it is unknown whether deceased donor kidney recipients have deliberately chosen for a deceased donor instead of approaching a living donor. Previous research among kidney patients on the waiting list showed that the most often mentioned first reaction in favour of deceased donor kidney transplantation was the unwillingness to burden a loved one, indicating fear of a decline in the donor’s health status [10]. In addition, psychological problems, particularly feelings of guilt and responsibility towards the donor were mentioned. With respect to relationship changes, this study reported that fear for inequality in the relationship after transplantation was present in many of the answers of kidney patients. However, as these patients were on the waiting list, these answers reflect their attitudes and not the impact of these attitudes on actual choices. More detailed information on the impact of relationship aspects on decision making to pursue living versus deceased donor kidney transplantation is thus lacking.

Purpose of the present study therefore was to explore motivations to donate or accept a living donor kidney or to pursue deceased donor kidney transplantation, and to assess whether expected relationship changes influence decision making and whether such relationship changes are actually experienced.

## Methods

### Participants

All donors and kidney recipients operated in the period 1997–2008 at the Leiden University Medical Center (LUMC) and who were still alive, were informed about the PARTNER study (Patients After Renal Transplantation and donation: long term Effects on health and Relationship). They were asked to respond if interested in focus group participation regarding possible relationship changes. Exclusion criteria were: insufficient command of the Dutch language, living abroad, severe psychological disorder (requiring treatment), anonymous donors and their recipients, donors / recipients from the crossover program, and recipients who lost their graft to create homogeneous groups. Based on the literature [6] and expert advice, it was considered that graft loss would influence the discussion to a great extent and would interfere with our goal to capture the views and motivations relevant for most of the study population.

Of the total population (n = 1097), 979 individuals were eligible to participate and a sample of 114 individuals was invited representative for the total study population with respect to age, gender and time since transplantation as the views of individuals may differ between e.g. younger and older individuals or by time passed since transplantation. These variables were available in the hospital information system and could thus be used for sampling. The focus groups were conducted separately for donors, living and deceased donor kidney recipients. The goal was to recruit equal numbers across the three groups with at least 6 participants and a maximum of 10 participants for each focus group to ensure everyone’s participation in the discussion. The study was approved by the LUMC Medical Ethics Committee.

### Focus groups

In June and July 2009, we conducted six focus group sessions lasting 1.5 to 2 h. Focus groups are a useful method to provide in-depth information and explore cognitions and motivations of underlying behavior [11,12]. We used the focus group procedures of Morgan and colleagues in preparing and conducting the sessions [13]. One moderator and two observers guided the sessions according to a carefully constructed protocol (Table 1), showing the topics that were addressed. These topics were based on literature review [6-10,14] and previously carried out face-to-face interviews with at least 3 people of each group (16 in total, not participating in the focus groups). These face-to-face interviews were carried out as a first in-depth exploration of motivations to donate or accept a kidney, and on the influence of relationship changes and examples of such changes. The topic list and questions shown in Table 1 were based on the issues where the interviews showed different views or we felt that further clarification was required (e.g. on how the relationship changed) before this could be translated into a questionnaire to the entire study population. The number of interviews per group was determined by saturation, meaning that no new major issues were raised, to ensure that all relevant topics were captured. 

**Table 1:** Focus group protocol

**Donors**	**Living donor kidney recipients**	**Deceased donor kidney recipients**
REASONS TO DONATE A KIDNEY AND REASONS TO PURSUE LIVING / DECEASED DONOR KIDNEY TRANSPLANTATION		
1. How did you obtain information on living kidney transplantation? *(awareness- knowledge)*	1. How did you obtain information on living kidney transplantation? *(awareness - knowledge)*	1. Were you aware of the possibility of living kidney transplantation? *(awareness – knowledge)*
2. Did someone ask you to donate a kidney or did you offer to donate a kidney? Were there several potential donors? *(efficacy)*	2. Did you ask someone to donate a kidney or did someone offer to donate a kidney? Were there several potential donors? *(efficacy)*	2. Were you afraid to ask or did you not want to ask someone to donate a kidney? Did you ask someone who refused? *(efficacy)*
3 What was the most important motivation to donate a kidney? *(attitude)*	3. What was the most important motivation to accept a kidney from this living donor and not to wait for a deceased donor? *(attitude)*	3. What was the most important motivation to pursue kidney transplantation with a deceased rather than a living donor? *(attitude)*
4. Did you experience social pressure to donate a kidney? *(social influences)*	4. Did you experience social pressure to accept a kidney? *(social influences)*	
RELATIONSHIP ASPECTS		
5. Did you expect that your relationship with the recipient would change after donation? Why (not)? *(predisposing factors - social)*	5. Did you expect that your relationship with the donor would change after the transplantation? Why (not)? *(predisposing factors - social)*	4. Did you expect changes in the relationship with a potential donor, and to what extent have these influenced the decision to wait for a deceased donor? *(predisposing factors - social)*
6. Has the relationship with the recipient changed after donation compared to before donation? How?	6. Has the relationship with the donor changed after transplantation compared to before transplantation? How?	

Individuals who consented to join the focus groups received a letter with the topics that would be discussed. Each topic contained specific questions and participants were asked to formulate their answers before the focus group sessions. During the sessions all participants wrote down their answers on post-its and posted these on a central board. This stimulated the discussion and enabled a quantitative assessment of how many participants made comments on a specific topic. The moderator ensured that the participants were prompted on all topics. The 2 observers took notes from the discussion, to ensure that the statements made on the post-its were correctly interpreted and classified into an appropriate category (see below). A quantitative count was added to support our qualitative analysis and to provide insight in the representativeness of the statements [15,16]. During the discussion, participants could put additional post-its on the board if additional arguments or issues were raised by the discussion.

The I-change model was used as a theoretical framework [17-19]. The rationale of the model is that for instance predisposing (social) factors determine awareness and a person’s attitude, social influence and efficacy, which in turn affect a person’s intention (motivation) to carry out certain behavior. Table 1 shows the relation between factors in the I-change model and the topics addressed during the focus groups. For instance, asking about social pressure relates to social influences in the I-change model that determine the intention to donate or accept a kidney. Efficacy was defined in this context as a person’s capacity to accept a kidney, so that questions were asked regarding the availability of potential donors.

### Statistical analysis

All focus group sessions were audio taped and transcribed in full. We analysed the transcripts using theory-based analysis, in which the text is organized according to pre-existing theoretical categories [20]. The transcribed text was coded into categories by two independent researchers (JD and PM), following the topics in the focus group protocol (related to the factors in the I-change model) using a software program for qualitative data analysis (Nvivo, version 8; QSR International; Doncaster, VIC, Australia) [20]. The notes from the observers were used to support appropriate analysis of the transcribed text into categories, particularly relevant in case of lively discussion by several participants (dis)agreeing with a statement on a post-it, which may not be fully captured by the audiotape.

We counted the number of participants who made comments fitting a specific category to support our qualitative analysis and to provide insight in the representativeness of the statements [15,16]. If participants made more than one similar comment on the same topic, these were counted only once. Descriptive and comparative statistics (Fisher exact test) were used to report and compare counts between donors and recipients from a living or deceased donor. Statistical testing was conducted to account for different group sizes, as a single opinion in one group may result in a higher percentage compared to another group. In this way, if statistically significant differences are found in opinions between these relatively small groups, we can expect that these will represent robust ‘meaningful’ differences.

A p-value smaller than 0.05 was considered significant in all analyses. Values between 0.05 and 0.10 were considered as a trend towards significance.

## Results

**Figure 1:**
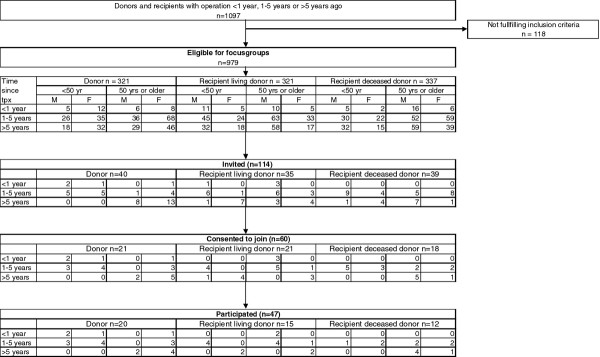
Flow diagram of focus group participants.

Of the 114 invited individuals, 60 (53%) consented to join the focus groups and 47 (41%) attended, representative for the total population with respect to age, gender and time since transplantation (Figure 1). The participation rate varied across the three groups between 31% and 50%, but was not significantly different (p = 0.22). Reasons for individuals not consenting were mostly that they did not want to participate in a focus group (n = 25), or could not attend because they were on a holiday during the time the focus groups were held (n = 14). Other reasons were: death of the recipient, feeling sick frequently, too busy or being abroad. Of the 60 individuals who agreed to attend, 13 did not participate of whom 3 cancelled shortly before the focus group due to health reasons. Table 2 shows characteristics of the participants (20 donors, 15 living and 12 deceased donor recipients). The groups did not differ in age, gender or time since transplantation and type of dialysis. As expected, the mean duration of dialysis before transplantation was longer among deceased donor kidney recipients (4 years vs. 0.5 years in living donor kidney recipients) consistent with waiting time estimates (http://www.eurotransplant.nl).

**Table 2:** Characteristics of participants

	**Donors** **(n = 20)**	**Living donor kidney recipients(n = 15)**	**Deceased donor kidney recipients(n = 12)**	**p-value**
Age, years (SD)	52.4 (12.2)	54.9 (9.1)	56.0 (10.9)	n.s.
Sex, No.				
Male	7 (35)	10 (67)	7 (58)	X^2^ = 3.78, p = 0.15
Female	13 (65)	5 (33)	5 (42)	
Time since surgery, years (SD)	3.7 (2.9)	3.7 (2.8)	4.8 (3.2)	n.s
Cause of renal failure in recipient^*^				
Diabetes	2	-	-	
Hypertension	-	-	1	
Glomerulonephritis	5	3	3	
Chronic pyelonephritis (reflux nephropathy)	2	3	1	
Cystic kidney	7	6	3	
Nephrocirrhosis	1	1	-	
Auto-immune cause (e.g. SLE)	2	-	1	
Malignancy	-	-	1	
Other urologic causes	1	1	-	
Unknown	-	1	2	
Type of dialysis in recipient^*^				
None	5	6	-	
Haemodialysis	6	4	4	X^2^ = 7.77, p = 0.20
Peritoneal dialysis	4	4	5	
Both	5	1	3	
Average duration of dialysis before transplantation in recipient, years (range) ^*^	1.2 (0–4.4)	0.5 (0–3.1)	4.0 (1.1–6.3)	p < 0.05
Donor-recipient relationship, No.				
Partner relationship	8	5		
Parent–child relationship (incl. in-law)	5	3	NA	
Brother-sister relationship (incl. in-law)	4	4		
Other family	1	-		
Friend	2	3		

NA = not applicable * For donors this refers to the recipient for whom they have donated a kidney.

### Predisposing factors

Expected donor-recipient relationship changes may relate to predisposing social factors and consequently affect individuals’ motivation to donate, accept or reject a kidney.

#### Donors

Donors mostly expected their relationship with the recipient to change positively; particularly that the recipient would be less dependent and participate more in family life (Table 3). Only one donor reported to fear some disbalance in the relationship; “If we have an argument after the transplantation, I may resent him for being angry at me while I donated a kidney for him!” (donor A9). The same donor also expected a positive change i.e., that the recipient would participate more in family life.

**Table 3:** Frequency of statements in the focus group sessions; differences between donors and living donor kidney recipients

**Statement categories**	**Donors (n = 20)**	**Living donor kidney recipients (n = 15)**	**p-value**
LIVING DONOR KIDNEY TRANSPLANTATION			
Information on transplantation with living donor			
Received information	3 (15)	4 (27)	0.43
Offer to donate			
Spontaneous offer to donate	9 (45)	13 (87)	**0.01**
Several donors present	6 (30)	9 (60)	0.08
Offer refused by recipient	2 (10)	2 (13)	1.00
Offer medically unfit for recipient	3 (15)	6 (40)	0.13
Offer withdrawn	1 (5)	1 (7)	1.00
Motivation to donate / accept / wait for deceased donor			
Disease progression / prospect of dialysis	9 (45)	2 (13)	0.07
Personal benefits	5 (25)	7 (47)	0.18
Other donors not possible	6 (30)	2 (13)	0.42
Anticipated regret	2 (10)		
Give chance to help		1 (7)	
Concern for donor’s health		3 (20)	
Relationship changes		4 (27)	
Social pressure			
Social pressure family	2 (10)	1 (7)	1.00
Social pressure doctors	5 (25)		
Internal (personal) pressure	4 (20)	1 (7)	0.37
RELATIONSHIP ASPECTS			
Expectations of relationship changes			
No relationship changes	3 (15)	7 (47)	0.06
Positive changes in donor-recipient relationship			
· Closer relationship	1 (5)	3 (20)	0.29
· Recipient less dependent / more participation in family life	5 (25)	1 (7)	0.21
· Having a normal life	3 (15)	1(7)	0.62
· Better quality of life of recipient	2 (10)		
Negative changes in donor-recipient relationship			
· Fear of imbalance in relationship	1 (5)	3 (20)	0.29
· Worries on relationship		1 (7)	
Experienced changes in relationship after surgery			
No changes in donor-recipient relationship	6 (30)	4 (27)	1.00
Positive changes in donor-recipient relationship			
· Having a normal life	2 (10)	3 (20)	0.63
· Recipient less dependent / more participation in family life	5 (25)	1 (7)	0.21
· Closer relationship	4 (20)	8 (53)	**0.04**
· More frequent contact / attention	2 (10)	1 (7)	1.00
Negative changes in donor-recipient relationship			
· Imbalance in relationship		4 (27)	
· Mood swings, improved quality of life less than expected	1 (5)		
· Sexuality, insecurity about scars	2 (10)	3 (20)	0.63
· Lack of recovery donor, many emotions, depression	2 (10)		
· Recipient wants to justify actions	2 (10)		
· Meddlesomeness donor (‘my kidney’) or others	4 (20)	2 (13)	0.68
· More frequent contact		1 (7)	
· Fear for remaining kidney / guilt feelings		3 (20)	
Changed relationship with others	7 (35)	7 (47)	0.49

Values are given as No. (%).

#### Living donor kidney recipients

Living donor kidney recipients mostly expected that their relationship with the donor would not change (Tables 3 and 4). If they expected a change, some were positive changes such as a closer relationship, while others expected negative changes such as fear for some imbalance in the relationship. One recipient for instant wondered “May I still argue with the donor? I was afraid not” (LD recipient B8) (Table 5). Another recipient expressed worries on the relationship: “What if my son breaks off his relationship with the donor? She still has donated a kidney for me” (LD recipient A2). Among the four recipients expecting negative changes, one recipient also expected positive changes.

**Table 4:** Frequency of statements in the focus group sessions; differences between living and deceased donor kidney recipients

**Statement categories**	**Living donor kidney recipients (n = 15)**	**Deceased donor kidney recipients (n = 12)**	**p-value**
DECEASED DONOR KIDNEY TRANSPLANTATION			
Information on transplantation with living donor			
Received information	4 (27)	7 (58)	0.13
Did not want information		1 (8)	
Offer to donate			
Spontaneous offer to donate	13 (87)		
Several donors present	9 (60)	6 (50)	0.60
Offer refused by recipient	2 (13)	4 (33)	0.36
Offer medically unfit for recipient	6 (40)	5 (42)	1.00
Offer withdrawn	1 (7)		
No (other) offer		2 (17)	
Did not want any (other) offer		6 (50)	
Motivation to donate / accept / wait for deceased donor			
Disease progression / prospect of dialysis	2 (13)		
Personal benefits	7 (47)		
Other donors not possible	2 (13)		
Give chance to help	1 (7)		
Young age of donor		6 (50)	
Concern for donor’s health	3 (20)	9 (75)	**0.04**
Obligation towards donor		10 (83)	
Disease is my responsibility		3 (25)	
Relationship changes	4 (27)	9 (75)	**0.01**
Social pressure			
Social pressure family	1 (7)		
Social pressure doctors			
Internal (personal) pressure	1 (7)		
RELATIONSHIP ASPECTS			
Expectations of relationship changes			
No relationship changes	7 (47)		
Positive changes in donor-recipient relationship			
· Closer relationship	3 (20)	1 (8)	0.61
· Recipient less dependent / more participation in family life	1 (7)		
· Having a normal life	1(7)		
Negative changes in donor-recipient relationship			
· Fear of imbalance in relationship	3 (20)	10 (83)	**0.01**
· Worries on relationship	1 (7)		
· Disappointment no offer		1 (8)	

Values are given as No. (%).

**Table 5:** Expressions of motivations to donate/ accept / wait for deceased donor, expected and experienced changes in relationship

**Statement category**	**Specific statements of participants classified in this category**
Motivation to donate / accept / wait for deceased donor	
· Concern for donor’s health	I was too afraid that my wife would not come out of the surgery so well (DD recipient A4)
· Relationship changes	I was afraid that in case of an argument the kidney would be brought up, even just as a joke. I didn’t want that (DD recipient A5)
· Personal benefits	I wanted to have a normal life again. In fact, the entire family was sick too (LD recipient B6)
	I did not want my children to see their father this way. I was prepared to go to great lengths for that (donor B7)
Social pressure	
· Social pressure doctors	The nephrologist said:"She needs a kidney transplantation as soon as possible, otherwise it is not necessary anymore, she will die without a kidney" (donor A11)
Expectations of relationship changes	
· Fear of imbalance in relationship	May I still argue with the donor? I was afraid not (LD recipient B8)
	I was afraid that after the transplantation it would be hard for me to say No to the donor (LD recipient B4)
	I was afraid I always had to be nice to the people who had given me a kidney (DD recipient A6)
Experienced changes in relationship after surgery	
· Closer relationship	The relationship is just as good, maybe even closer because we share this together (LD recipient B5)
	We’re closer than we used to be before the transplantation: it just happened (donor B4)
· Imbalance in relationship	I struggle with the balance. I do not dare to say No to the donor as I would want to do (LD recipient B4)
	I have the feeling I have to be grateful all my life (LD recipient A4)
	I feel obligated to maintain a good relationship with the donor, while she is not my type (LD recipient A5)
· Meddlesomeness donor	Once in the month: did you take your medication? (donor A4)
	It is spare time for the recipient so be careful with yourself (donor A8)
· Changed relationship with others	The relationship with the partner of the recipient also improved (donor B4)
	The mutual relationship of the children has become closer in the period that both their parents were sick (donor A7)
	We were disappointed that nobody in the family offered to donate a kidney. We try to still maintain a good relationship (donor B6)

#### Deceased donor kidney recipients

Most deceased donor kidney recipients (83%) were afraid of imbalance in the donor-recipient relationship (Table 4). One recipient stated “I was afraid I always had to be nice to the person who had given me a kidney” (DD recipient A6). Others stated: “So you cannot say the truth the way you think about it” (DD recipient A4) or “I would not dare to say No anymore because of having received a kidney” (DD recipient B4). These fears regarding expected relationship changes were reported more often by deceased than living donor kidney recipients (p = 0.01). Most deceased donor kidney recipients expected negative changes; only one expected positive changes as well, while most living donor kidney recipients expected only positive changes. Thus, relatively more deceased donor kidney recipients mainly expected negative relationship changes, while living donor kidney recipients expected positive changes besides some negative expectations.

### Awareness

To decide on kidney donation or acceptance, people need to be aware that living donor kidney transplantation is possible, and thus should have received information. All participants were aware that living donor kidney transplantation was possible, and had received the necessary information (Table 3 and 4). Only one deceased donor kidney recipient stressed not wanting any information on this topic.

### Motivational factors

Efficacy, attitude and social pressure are motivational factors that may determine a person’s intention to donate or accept a kidney.

#### Efficacy

Several donors were often available, similarly in living and deceased donor kidney recipients (Table 4). The offer to donate was sometimes refused, or was not suitable for the recipient for medical reasons, similar in both groups (Table 4). So the reason to pursue deceased donor kidney transplantation is not always the lack of a potential donor. Only 1 deceased donor kidney recipient (8%) did not have a potential living donor. However, he had received information on living donor kidney transplantation and considered asking someone to donate a kidney or discussing this option within the family.

#### Attitude

#### Donors

Donors mostly offered a kidney motivated by the progression of the recipients’ disease and/or the prospect of dialysis in the (near) future (45%). However, they also stressed their personal benefits in donating a kidney; they hoped the transplantation would make the recipient less dependent and thus could participate more in household activities and family life (Table 3).

#### Recipients

As expected, living donor kidney recipients mostly accepted the kidney motivated by the expected improvement of their quality of life (personal benefit) (Tables 3 and 4).

Deceased donor kidney recipients on the other hand, often waited for a deceased donor kidney because of feelings of obligation towards the donor (83%) (Table 4). Another motivation was their concern for the donor’s health, which was reported more often than by living donor kidney recipients (75% vs. 20%, p = 0.04). They also reported fear of relationship changes more often than living donor kidney recipients (75% vs. 27%, p = 0.01). So the motivations among deceased donor kidney recipients to wait, are partly shared by living donor kidney recipients but apparently are less pronounced or decisive, since the latter group decided to pursue living kidney transplantation despite their fears.

#### Social pressure

In all groups, a minority experienced some social pressure. 10% of the donors experienced social pressure by family members (Table 3) and 25% experienced social pressure by doctors. Donors reported statements by doctors like “She needs a kidney transplantation as soon as possible; otherwise it is not necessary anymore. She will die without a kidney” (donor A11) (Table 5). Donors also mentioned internal pressure they placed on themselves to offer a kidney (Table 3). Living donor kidney recipients less often experienced social pressure by family members (7%) or internal pressure (7%) to accept a kidney (Table 3).

### Experienced relationship changes after living kidney transplantation

About one-third of both donors and recipients reported no change in their relationship after transplantation (Table 4). Those with recent surgery (<1 year after transplantation) were more likely to report no changes in the relationship (p < 0.01) than those 1–5 years or more than 5 years after transplantation, particularly in recipients. Compared to their expectations before transplantation, more donors and recipients reported a closer relationship after transplantation. An experienced closer relationship was reported more often by recipients than by donors (53% vs. 20%, p = 0.04). One recipient stated “The relationship is just as good, maybe even closer, because we share this together” (LD recipient B5). However, both groups also experienced some unanticipated negative relationship changes such as the donor being meddlesome regarding ‘his/her kidney’ (20%), or fear for the donor’s remaining kidney and feelings of guilt (20%). Some recipients actually experienced imbalance in their relationship with the donor (27%): “I struggle with the balance. I do not dare to say No to the donor as I would want to” (LD recipient B4), “I have the feeling I have to be grateful all my life” (LD recipient A4), “I feel obligated to maintain a good relationship with the donor, while she is not my type” (LD recipient A5) (Table 5). Among the 14 donors experiencing negative changes, 10 also experienced positive changes. Among 12 recipients experiencing negative changes, 11 also experienced positive changes. So even though negative changes may occur, these seem to be counterbalanced by positive changes.

An unanticipated finding in all focus groups was that both donors (35%) and recipients (47%) stressed that relationship changes also involved other people (e.g. the partner of the recipient, children or brother/sisters) (Table 3). These changes were considered both positive and negative (Table 5).

## Discussion and conclusion

The present study has shown that deceased donor kidney recipients were aware that living donor kidney transplantation was possible. They often had a potential donor available which they refused or did not want. They mostly waited for a deceased donor because of their concern about the donor’s health. They more often expected negative relationship changes than living donor kidney recipients, who also expected positive changes. Living donor kidney recipients mostly accepted the kidney to improve their own quality of life, combined with expected mostly positive relationship changes. Donors mostly donated a kidney because transplantation would make the recipient less dependent and could participate more in family life, thereby improving the donor’s quality of life.

A limitation of our study is that we counted only verbal statements made in the focus groups, without taking into account the nonverbal expressions (e.g., nodding agreement to statements of other participants) [20]. Nevertheless, the quantitative counts of verbal utterances support our impressions from all focus groups. A second limitation is that we asked respondents retrospectively about their motivations and expectations prior to transplantation. Not all donors and recipients may remember their motivations or feelings prior to transplantation exactly, so that recall bias could result in over-representation of strong emotions that are still remembered. Our results may also be biased due to cognitive dissonance: people tend to justify earlier decisions, resulting in other motivations or emotions being reported than in a prospective study. A third limitation concerns the selection of participants. We may have observed the opinions of a selected group willing to participate in our study, e.g. because they had expected or experienced relationship changes. This may overestimate the percentage of persons reporting relationship changes. However, it is unlikely that this selection will have biased the reported specific aspects of the relationship changes or influenced differences between the three groups. Thus, counting responses, as done within this study, gave a good impression of the important key themes. Further research should show whether our results apply to a larger group of donors and recipients. We will translate the most frequently reported relationship changes and motivations to donate or accept a kidney into questions for a questionnaire sent to our entire study population. In this way we will obtain quantitative estimates on what percentage of donors and recipients experienced relationship changes, whether donors and recipients have the same views on these changes, and what may be possible determinants of such relationship changes.

Our study is, to our knowledge, the first that explored which factors influence patients in their decision making regarding living or deceased donor kidney transplantation with both qualitative and quantitative methods. This combination of methods enables us to conclude that certain types of motivations, expectations and fears seem more common than others. At the same time we could show the inter-individual variation in the precise motivation regarding living kidney transplantation. Previous studies showed that the main motivating factors for donors are the stress and anxiety of subjecting ‘a loved one’ to prolonged dialysis therapy and the emotional and physical deterioration associated with long-term dialysis therapy [6,21]. Binet et al. showed that the donation was based on indirectly gained benefits for themselves through the improvement of the recipient’s condition [22]. This is consistent with our results where donors also reported disease progression and personal benefits as important reasons to offer a kidney. Living donor kidney recipients also mentioned personal benefits as a reason to accept a kidney. However, these motives seemed less important among deceased donor kidney recipients, who waited for a deceased donor kidney motivated by the feeling they would otherwise have an obligation towards the donor. Moreover, they are concerned about the donor's health consistent with the results found by [Waterman et al. 23], which was reported less often by living donor kidney recipients. The present study adds a direct comparison between living and deceased donor kidney recipients. Thus, the motivation behind living kidney transplantation varies not only from one individual to another but also between groups of kidney patients affecting their decision to pursue living or deceased donor kidney transplantation.

Living and deceased donor kidney recipients also differed in their expectations regarding donor-recipient relationship changes. Most previous studies regarding the effect of living kidney donation on the donor-recipient relationship have been performed from the donor’s perspective [7,8]. Some studies reported no change, while others reported an improved donor-recipient relationship [7,24-32]. If a more detailed description of the donor-recipient relationship after transplantation was given, it was often defined as stable or close [24,26,28]. The present study includes both perspectives and shows that expected relationship changes differ, which has determined the motivation to donate, accept or refuse a kidney. Living donor kidney recipients expect both negative changes such as a fear of some imbalance in the relationship and positive changes, such as a closer relationship. Deceased donor kidney recipients expected the same negative changes, but did not expect positive changes. Kranenburg et al. also found that kidney patients on the waiting list expected and feared an unequal, disturbed relationship with the donor after transplantation [10], but did not compare expectations or experiences of living versus deceased donor kidney recipients. Our results indicate that fear of donor-recipient relationship changes influences the decision of patients not to pursue living donor kidney transplantation either by refusing an offer or by stating not to want any offer. The present study also shows that negative relationship changes are indeed experienced to some extent, but that positive changes are experienced more often even though not always expected beforehand. These changes are a closer relationship and increased participation of the recipient in family life. It seems important to include this in the information given to future (potential) donors and recipients. About one-third of our participating donors and recipients experienced no change in the relationship after transplantation, especially those who underwent surgery recently. Even though we have to be careful given the small numbers, a potential explanation may be that it takes some time before realizing that something has changed, particularly if the changes are subtle. Given the small numbers, we cannot make any comparisons on specific positive or negative relationship changes that are experienced, but this will be possible in our future questionnaire study.

Surprisingly, we found that deceased donor kidney recipients often had a potential donor available, but that this offer was refused. These recipients chose to wait for a deceased donor kidney, because of their concern for the living donor’s health and expected negative relationship changes without any positive expectations. It is important to identify these kidney patients to address these issues and to take away any unjustified fears. This may be achieved by discussing expectations regarding changes in the relationship and their health status, as an element of standard care. If these issues are only discussed when brought up, things may be left unsaid so that the potential donor or recipient is not aware of these fears or expectations. They should at least feel reassured they can discuss their fears and doubts regarding living donor kidney transplantation; stories of previous recipients may help in this situation, as well as evidence on how many donors or recipients have actually experienced such changes. By making it part of the standard set of questions, it becomes clear that these issues are just as important as questions on medical issues and need to be considered. In this way, they are prepared what might happen after transplantation and are supported in their decision making.

Most previous studies report that only a very small percentage of donors perceived external pressure to donate a kidney, with estimates in the range of 5–10% [30,32,33]. The percentage of donors experiencing social pressure, by either family or physicians, seems higher among our participants. However, given the small numbers, we have to be careful in interpreting these estimates given that a single answer may have a considerable influence on the resulting estimate, and the fact that the focus group may have been a selective sample. On the other hand, if it were true it may possibly be explained by the fact that more subtle changes were picked up in the focus groups than in previous questionnaire studies. This is supported by a recent study of Valapour et al. who asked donors to rank the extent of pressure on a 5-point scale and reported that 40% of donors felt some pressure to donate, with only 2% reporting the highest social pressure [34].

Whether these results can be generalized to other centers will probably depend on differences in cultural values, health care policies and waiting list systems. For instance, Martinez-Alarcón et al. have shown that the general attitude towards living versus deceased donor kidney transplantation is different in Spain, where the majority of patients prefers to wait for a deceased donor, most likely explained by the shorter waiting time in Spain compared to the Netherlands [35]. Another explanation may be the reluctance of transplant professionals to offer living kidney donation systematically to all patients even though they have a general positive attitude towards living kidney donation [36]. These issues are likely to influence the extrapolation of our findings to other countries. We do not expect large differences in the attitude of patients within the Netherlands, given for instance the national waiting list, or large differences in the attitude of transplant professionals, so that we expect that our findings can be generalized to other Dutch centers.

In conclusion, fear of donor-recipient relationship changes and concerns about the donor’s health seem more substantial in deceased donor kidney recipients, resulting in a decision to wait for a deceased donor despite having a potential living donor available. Further research is needed to assess whether this concerns a particular group of recipients and whether it is possible to eliminate their fears or take it into account in their decision making. If confirmed, information prior to living donor kidney transplantation should address expectations regarding potential relationship changes.

## Competing interests

The authors declare that they have no competing interests.

## Author’s contributions

IBG analyzed data and wrote the first draft of the manuscript; KS guided the focus groups, participated in study design and interpretation of the data; SD, PJMB, AMS and AGB helped with interpretation of the data and writing the manuscript; PJM conceived the study and its design, participated in the statistical analyses and interpretation of the data, and writing the manuscript. All authors have read and approved the final manuscript.

## Funding

This study was funded by the Dutch Kidney Foundation, program Patient Care.

## Pre-publication history

The pre-publication history for this paper can be accessed here:

http://www.biomedcentral.com/1471-2369/13/103/prepub
